# Therapeutic Mechanism and Effect of Camptothecin on Dextran Sodium Sulfate-Induced Ulcerative Colitis in Mice

**DOI:** 10.1155/2021/5556659

**Published:** 2021-04-24

**Authors:** Yizhuo Wang, Kunjian Liu, Zhiyong Qi, Tong Chen, Wei Yu, Yang Jiang, Guofeng Li, Huijie Xiao

**Affiliations:** ^1^Department of Cancer Center, First Hospital of Jilin University, Changchun 130000, China; ^2^Department of Anorectal, Affiliated Hospital of Changchun University of Traditional Chinese Medicine, Changchun 130021, China; ^3^Department of Gastrointestinal Colorectal and Anal Surgery, China-Japan Union Hospital of Jilin University, Changchun 130033, China

## Abstract

Camptothecin (CPT) is a cytotoxic quinoline alkaloid isolated from the bark and branches of the Chinese tree *Camptotheca acuminata*. CPT inhibits topoisomerase I. It possesses various antitumor activities and is mainly used in the treatment of colon, ovarian, liver, and bone cancers as well as leukemia. CPT inhibits the expressions of inflammatory genes and can prevent death from chronic inflammation. Therefore, we investigated the effect of CPT treatment in ulcerative colitis (UC) using DSS-induced UC mouse model; after that, we explored its potential mechanisms. Here, we found that CPT exerted protection on DSS-induced UC in rats. In addition, the administration prominently reduced the disease activity index as well as colon length of the model rats and remarkably reduced the inflammatory cytokines. Further, CPT significantly reduced several vital proinflammatory proteins in LPS-induced RAW264.7 cells. In summary, our findings demonstrate that CPT is hopefully to act as a therapeutic agent for UC.

## 1. Introduction

Ulcerative colitis (UC) is an inflammatory disease of the colonic mucosa, which is usually chronic. It originates in the rectum and usually extends downwards in a continuous manner, through one part of the colon or through the whole colon. However, some patients have an inflammation of the cecal patch. A typical symptom of UC is bloody diarrhea. The clinical courses are unpredictable and its characteristic is deteriorating, and mitigation occurs alternatively [1]; it has spontaneous changes or responds to treatment [2]. Although the etiology of UC is still unclear, there is an international consensus that inflammation in genetically susceptible individuals is caused by impaired intestinal mucosal immune regulation caused by multiple environmental factors [3]. Abundant studies have shown that UC is mediated by T helper type 2-produced cytokines and is a disorder associated with increased IL-6 and at the same time decreased IL-10 levels. These proinflammatory factors are crucial in the pathogenesis of UC [4, 5]. Therefore, a large number of therapeutic drugs that suppress inflammatory response are applied in treating UC, for instance, 5-aminosalicylate and glucocorticoids [6], but the overall effect is limited. Relapse is common after treatment discontinuation, and there are many adverse reactions. Consequently, there is patient demand for complementary and alternative drugs with fewer side effects.

**Figure 1:**
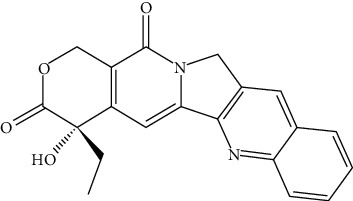
Structure of camptothecin.

Camptothecin (CPT) isolated from the bark and branches of the *Camptotheca acuminata*, a kind of Chinese tree, is a natural product. This cytotoxic quinoline alkaloid (Figure 1) inhibits topoisomerase I and exerts a broad spectrum of antitumor activity; it is mainly used to treat colon, ovarian, liver, bone, and small lung cancers as well as leukemia [7]. For example, in the process of treating liver cancer, Li et al. used lysine-mediated hydroxyethyl starch-10-hydroxy CPT micelles. Although many studies have identified CPT as an anti-inflammatory drug [8], there are few anti-inflammatory studies. In 2016, Rialdi et al. reported that CPT inhibited the expressions of inflammatory genes and prevented death from chronic inflammation [9]. But the effect of CPT administration on UC has not been investigated.

Numerous studies have shown that AKT/NF-*κ*B and MAPK signaling pathways are indispensable in inflammatory response [10, 11]. Activation of these signaling pathways has been observed to promote and inhibit inflammatory responses, whereas the inhibition of their activation can suppress inflammatory response which has become the focus of follow-up research.

We studied CPT as a natural preparation for the treatment of UC and explored its effect on dextran sodium sulfate-induced UC in a model and LPS-induced RAW264.7 cells to identify alternative treatments for UC.

## 2. Materials and Method

### 2.1. Drugs and Reagents

CPT (purity > 98%) was obtained from Chengdu Pufei De Biotech Co., Ltd. (Sichuan, China). Dimethyl sulfoxide, fetal bovine serum, modified Eagle medium, ELISA kits, and CCK8 kit all have common sources as the standard recommended. Resorcinol, H_2_O_2_, and Hepes were bought from Sigma Chemical Co. (USA). The antibodies, COX-2 and iNOS, occluding and *β*-acting came from specialized corporation. Donkey anti-rabbit or anti-mouse IgG and goat anti-mouse and goat anti-rabbit secondary antibodies were all purchased.

### 2.2. Cell Culture

RAW264.7 cells were purchased from the American Type Culture Collection (ATCC® CRL-3063™) and cultured in DMEM containing 10% FBS at 37°C in a humidified incubator under 5% CO_2_.

### 2.3. Cell Viability

The CCK8 method was applied to detect the influence of CPT against cell activity. RAW264.7 cell was disposed of CPT for 24 hours of different doses (0.1, 0.2, 0.4, 0.6, 0.8, 1.0, 2.0, and 5.0 *μ*mol/mL). After that, add processing of 10 *μ*L CCK8. Then, measure optical density after 3 hours.

### 2.4. Animal Experimental Design

All the 8-week C57BL/6 mice were purchased from Liaoning Changsheng Biotechnology Co., Ltd. In order to explore the alleviative effect of CPT on colitis in mice, we divided the mice into six groups: control group, CPT group, DSS group, DSS+CPT group (0.5 mg/kg), DSS+CPT group (1 mg/kg), and DSS+CPT group (1.5 mg/kg). During the experiment, mice were free to eat and drink water. Three days before the experiment, mice in the CPT group were fed with CPT (0.5 mg/kg, 1 mg/kg, and 1.5 mg/kg). At the beginning of the experiment, mice in the DSS group and the DSS+CPT group were fed with 2% DSS water, and CPT was continually fed every day.

### 2.5. Index of Disease Activity

Weight and fecal characteristics including occult blood were observed during this experiment. The scoring system described by Tian et al. was then used to calculate the disease activity index [12] (Table 1).

### 2.6. ELISA for TNF-*α*, IL-6, and IL-1*β*

Protein levels of TNF-*α*, IL-6, and IL-1*β* in colon tissue samples were detected by ELISA kits according to technical manual.

### 2.7. H&E Staining

We collected the colon of mice for tissue fixation and H&E staining. The staining method and the disease activity index in total were conducted according to Guo et al. [13].

### 2.8. MPO Activity Determination

Colon tissue samples were gathered, ground, and then centrifuged as the same treatment as Liu et al. [14].

### 2.9. Immunofluorescence Assays

The cultured cells were separated into four groups with four cells per group. We discarded the medium, used PBS to wash the cells for 3 times in 15 min, added 1 mL of immunostaining fixative solution (p0098, Beyotime, Shanghai, China) in each well, and incubated the microplate for 10 min at 25°C, permeabilizing cell membranes with 1% (*v*/*v*) Triton X-100 and washing 3 times in 15 min. Following the addition of 5% PBS-diluted goat serum, seal the wells and then incubate those 3 hours at 25°C. After that, discard the blocking solution and incubate cells for one night. When three times (5 min/wash) of PBS washing were done, fluorescent secondary antibody diluted with 5% goat serum (1 : 1000) was added and these cells were incubated at 25°C under darkness for an hour. When three times (5 min/wash) of PBS washing were done, it was sealed with a sealant containing 4′,6-diamidino-2-phenylindole. A laser confocal microscope was used to observe and record cell images.

### 2.10. qRT-PCR Analysis

Extract total RNA from cultured RAW264.7 cells and then apply qRT-PCR to detect the expression levels of target protein. *β*-Actin was applied to normalize gene expression.  Table 2 shows the primer sequences.

### 2.11. Western Blot

The RIPA lysate was used to extract the total protein from RAW264.7 cells and mouse colon samples, and the BCA protein concentration assay kit was used to detect the protein concentration. Western blot was performed according to Guo et al.'s procedure [15].

### 2.12. Statistical Analysis

GraphPad Prism 7.0 was applied in performing analyses statistically and generating images presenting the results. Mice were separated to groups randomly. Histological analysis was performed. In the case of significant population *F*-test (*P* < 0.05), Tukey's adjustment method was used for special comparison.

## 3. Results

### 3.1. CPT Administration Reduces Pathological Damage of Colon Tissue in Mice with DSS-Induced US

**Figure 2:**
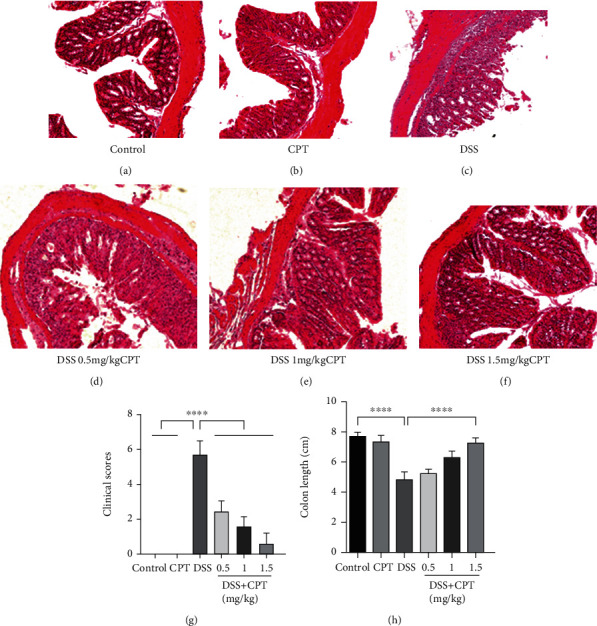
Effects of camptothecin (CPT) on the pathological injury of colon tissues in mice with DSS-induced ulcerative colitis (UC). Colon tissue samples were collected from the (a) control group, (b) CPT (1.5 mg/kg) group, (c) DSS group, (d) DSS+CPT (0.5 mg/kg) group, (e) DSS+CPT (1.0 mg/kg) group, and (f) DSS+CPT (1.5 mg/kg) group and stained with H&E. (g) Disease activity index and (h) colon length of the different groups. The mean disease activity index of colon tissues was determined according to a previously described three-point scale. Values are presented as means ± SD (^∗∗^*P* < 0.01 and ^∗∗∗∗^*P* < 0.0001 vs. the DSS group).

Colon tissue sections were used for hematoxylin-eosin staining with the purpose to study the protection of CPT (Figure 2). The DSS-induced UC mice exhibited an obvious induction effect, compared with the control group, which was characterized by increased colon injury, significantly reduced levels of goblet cells, increased cell infiltration, mucous membrane edema, and structural damage of the colon (Figures 2(a) and 2(c)). But CPT reduced these pathological lesions and it is dose-dependent (Figures 2(c)–2(f)). The index of disease activity (Figure 2(g)) and mouse colon length (Figure 2(h)) results suggested that, in DSS-induced mice, CPT dose-dependently reduced the activity index of disease as well as length of colon.

### 3.2. CPT Inhibits Myeloperoxidase (MPO) Activity in Mice with DSS-Induced UC

**Figure 3:**
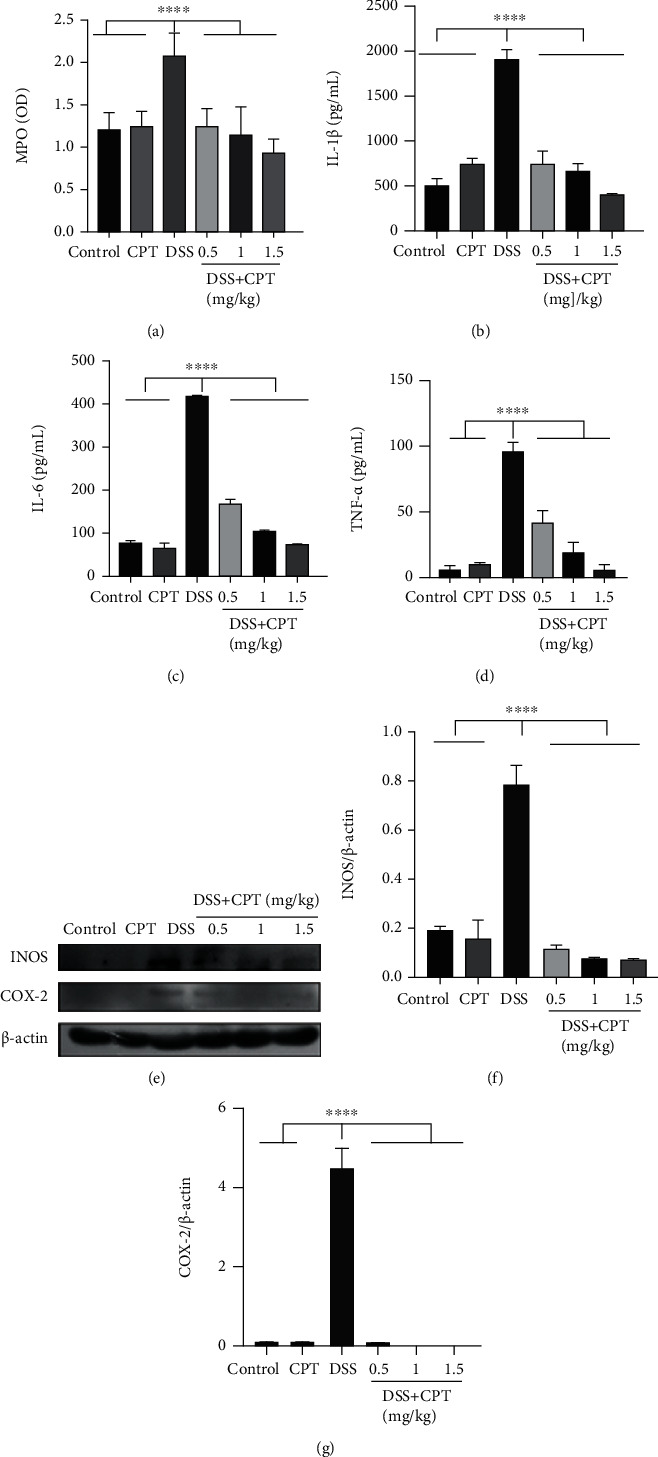
Effects of camptothecin (CPT) on the inflammatory response of DSS-induced ulcerative colitis (UC) in mice. (a) Myeloperoxidase (MPO) activity assay. The protein levels of (b) IL-1*β*, (c) IL-6, and (d) TNF-*α* were detected using ELISA. Western blot assay of (e, f) inducible nitric oxide synthase (iNOS) and (e, g) cyclooxygenase-2 (COX-2). The relative protein levels were quantified by scanning densitometry and normalized to the protein level of *β*-actin. Values are presented as mean ± SD (^∗^*P* < 0.05, ^∗∗^*P* < 0.01, and ^∗∗∗∗^*P* < 0.0001 vs. the DSS group).

The activity of MPO was determined by us and used as a neutrophil marker to examine the inflammation degree. MPO activity in the DSS group was remarkably improved (Figure 3(a)). Compared with the DSS group, the CPT groups dose-dependently showed significantly reduced MPO activity (Figure 3(a)).

### 3.3. CPT Reduced the Release of Inflammatory Mediators in Mice with DSS-Induced UC

Proinflammatory cytokines are indispensable in the inflammation development. We determined the levels of three major proinflammatory cytokines in colon tissues by ELISA to demonstrate the CPT effect on inflammation level induced by DSS in UC mice. In the DSS group, the IL-1*β* level (Figure 3(b)), IL-6 level (Figure 3(c)), and TNF-*α* level (Figure 3(d)) all remarkably increased, whereas CPT treatment inhibited this effect. We detected the levels of two vital proinflammatory protein, iNOS and COX-2, associated with inflammation by western blot. The DSS group showed significant induction of the production of these two vital proteins (Figures 3(e)–3(g)) in the colon, whereas the CPT groups showed significant inhibitory effect on colonic products of UC mice induced by DSS.

### 3.4. CPT Inhibits the AKT/NF-*κ*B and MAPK Signaling Pathways in Mice with DSS-Induced UC

**Figure 4:**
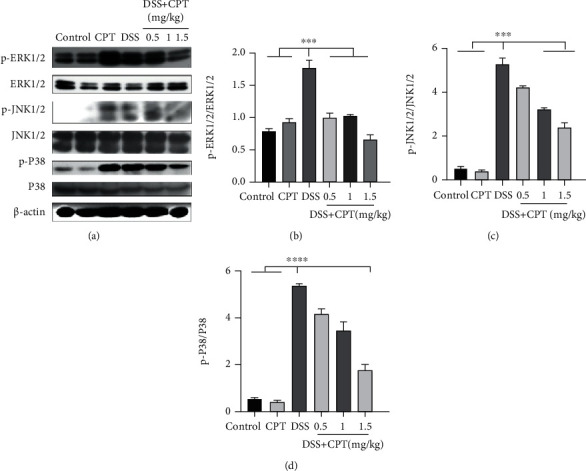
Effects of camptothecin (CPT) on the MAPK signaling pathway in DSS-induced ulcerative colitis in mice. Tissue lysates were prepared and subjected to western blot using (a, b) p-ERK1/2, (a, c) p-JNK1/2, and (a, d) p-P38 antibodies. The p-ERK1/2, p-JNK1/2, and p-P38 immunoreactive bands were digitized and expressed as a ratio of ERK1/2, JNK1/2, and P38 levels (b–d). Values are presented as mean ± SD (^∗∗∗∗^*P* < 0.0001 vs. the DSS group).

**Figure 5:**
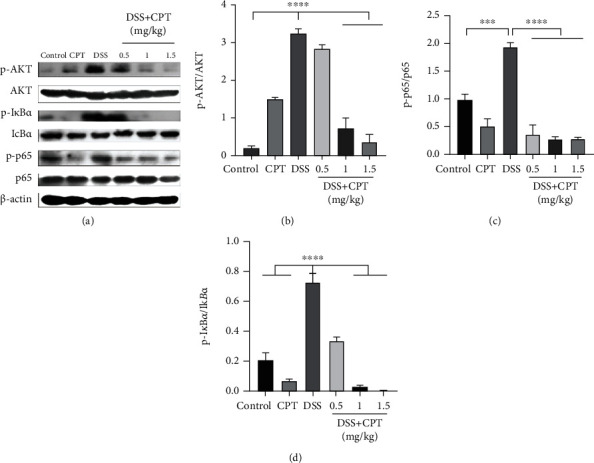
Effects of camptothecin (CPT) on the AKT/NF-*κ*B signaling pathway in DSS-induced ulcerative colitis in mice. Tissue lysates were prepared and subjected to western blot using (a, b) p-AKT, (a, c) p-I*κ*B*α*, and (a, d) p-p65 antibodies. The p-AKT, p-p65, and p-I*κ*B*α* immunoreactive bands were digitized and expressed as a ratio of AKT, I*κ*B*α*, and p65 levels (b–d). Values are presented as mean ± SD (^∗^*P* < 0.05, ^∗∗∗^*P* < 0.001, and ^∗∗∗∗^*P* < 0.0001 vs. the LPS group).

The AKT/NF-*κ*B and MAPK signaling pathways are found to be vital producing mediators of inflammation. We examined the protein expressions of these signaling pathways by western blot to prove its anti-inflammatory characteristic. The extracellular phosphorylation signal regulatory protein level in the DSS group, that is, ERK1/2 (Figures 4(a) and 4(b)), JNK1/2 (Figures 4(a) and 4(c)), P38 (Figures 4(a) and 4(d)), AKT (Figures 5(a) and 5(b)), inhibitor of nuclear factor-*κ*B (I*κ*B*α*; Figures 5(a) and 5(d)), and p65 (Figures 5(a) and 5(c)), was remarkably higher. Additionally, the CPT groups showed significant dose-dependent inhibition of DSS-induced phosphorylation of ERK1/2 (Figures 4(a) and 4(b)), JNK1/2 (Figures 4(a) and 4(c)), P38 (Figures 4(a) and 4(d)), AKT (Figures 5(a) and 5(b)), I*κ*B*α* (Figures 5(a) and 5(d)), and p65 (Figures 5(a) and 5(c)).

### 3.5. CPT Inhibits the LPS-Induced Inflammatory Response of RAW264.7 Cells

**Figure 6:**
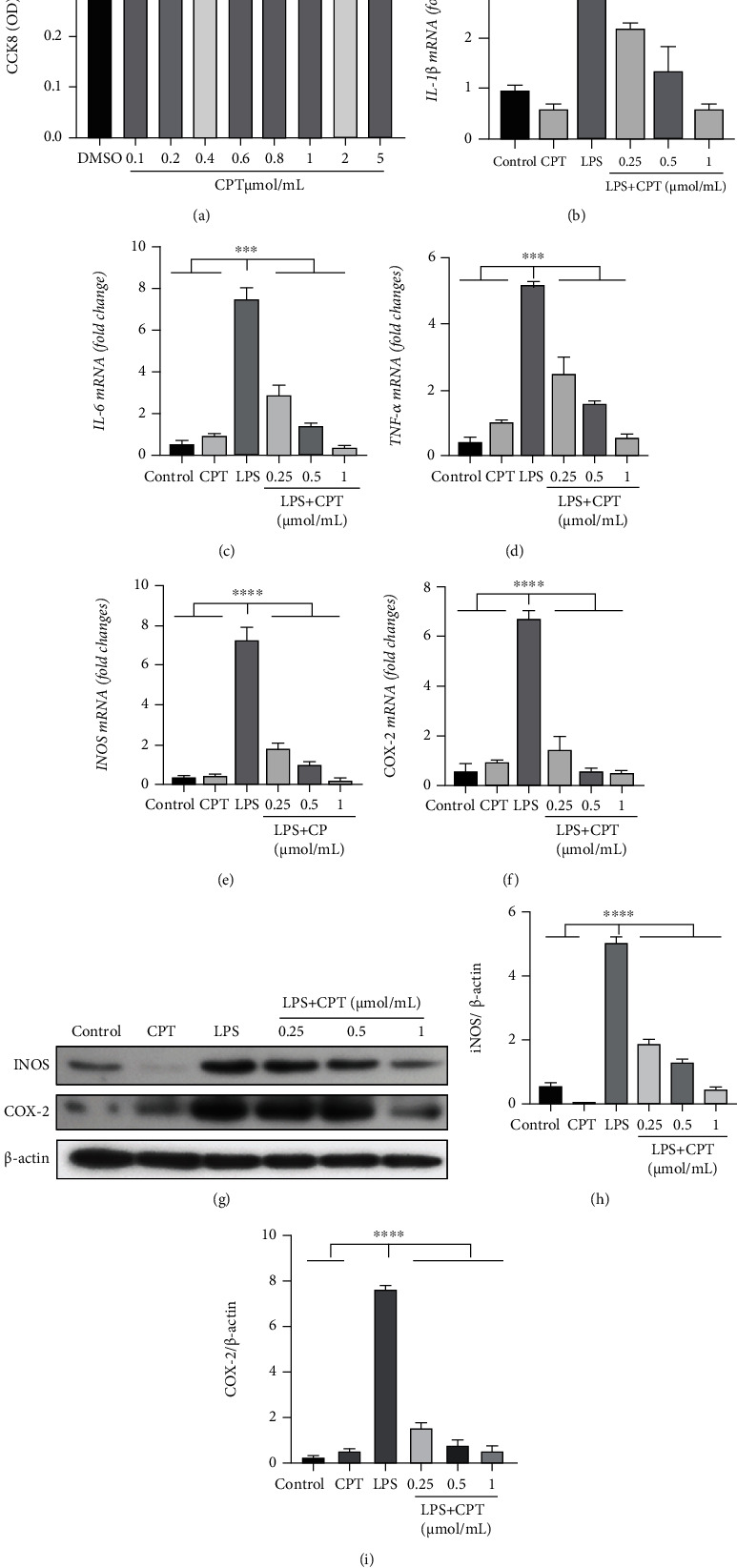
Effects of camptothecin (CPT) on LPS-induced inflammatory response in RAW264.7 cells. RAW264.7 cells were cultured with different doses of CPT (0.1, 0.2, 0.4, 0.8, 1.0, 2.0, and 5.0 *μ*mol/mL) for 4 h, and their viability was determined by CCK8 assay. (a) The effect of CPT was determined by CCK8 assay. RAW264.7 cells were pretreated with different doses of CPT (0.25, 0.5, and 1 *μ*mol/mL) for 1 h and stimulated with LPS for 4 h. Protein and mRNA levels were determined by qRT-PCR and western blot, respectively. The mRNA levels of (b) IL-1*β*, (c) IL-6, (d) TNF-*α*, (e) iNOS, and (f) COX-2 were determined and normalized to the mRNA level of *β*-actin. The protein levels of (g, i) COX-2 and (g, h) iNOS and the relative protein levels were quantified by scanning densitometry and normalized to the protein level of *β*-actin. Values are presented as mean ± SD (^∗^*P* < 0.05, ^∗∗^*P* < 0.01, ^∗∗∗^*P* < 0.001, and ^∗∗∗∗^*P* < 0.0001 vs. the LPS group).

Our in vivo studies showed that the effect CPT caused in DSS-induced UC is anti-inflammatory. Therefore, we investigated CPT on LPS-induced RAW264.7 cell in terms of inflammatory response and then illustrate the anti-inflammatory function. We used CCK8 assay to detect the cytotoxicity on RAW264.7 cells from CPT. CPT was not toxic to RAW264.7 cells at a dose of 0.1–1.0 *μ*mol/mL (Figure 6(a)). We also pretreated RAW264.7 cells for an hour with CPT (0.25, 0.5, and 1.0 *μ*mol/mL) and then excited them for 4 hours using LPS (1 *μ*g/mL). We took advantage of qRT-PCR to examine the mRNA levels of several vital proinflammatory proteins. After that, we applied western blot to test the expression levels of these proteins. And the results showed that the mRNA levels were remarkably higher in the LPS group (Figures 6(b)–6(f)), whereas the CPT groups showed significant dose-dependent inhibition of the mRNA levels (Figures 6(b)–6(f)). The western blot results exhibited that CPT inhibited the level of LPS-induced protein, namely, COX-2 and iNOS. The effect was noteworthy and dose-dependent (Figures 6(g)–6(i)). These results are in agreement with our in vivo results, which verified the CPT's anti-inflammatory effect.

### 3.6. CPT Inhibits the Activation of the AKT/NF-*κ*B and MAPK Signaling Pathways in RAW264.7 Cells Stimulated by LPS

**Figure 7:**
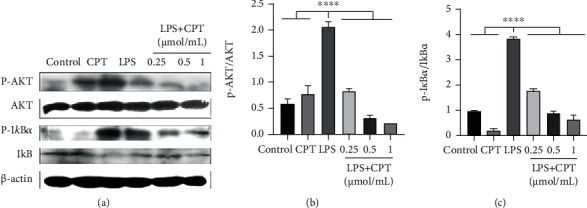
Effects of camptothecin (CPT) on LPS-induced activation of the AKT/NF-*κ*B signaling pathway in RAW264.7 cells. Total protein in RAW264.7 cells was collected following 4 h of LPS stimulation. CPT was added 1 h before LPS stimulation. Protein levels of p-AKT and p-I*κ*B*α* were detected by western blot and quantitatively assessed via densitometry using (a, b) AKT and (a, c) I*κ*B*α* as internal controls. Protein levels were measured using ImageJ (http://imagej.nih.gov/ij/) and normalized to the protein level of AKT and I*κ*B*α*. Values are presented as mean ± SD (^∗∗^*P* < 0.01 vs. LPS, ^∗∗∗∗^*P* < 0.0001 vs. LPS).

**Figure 8:**
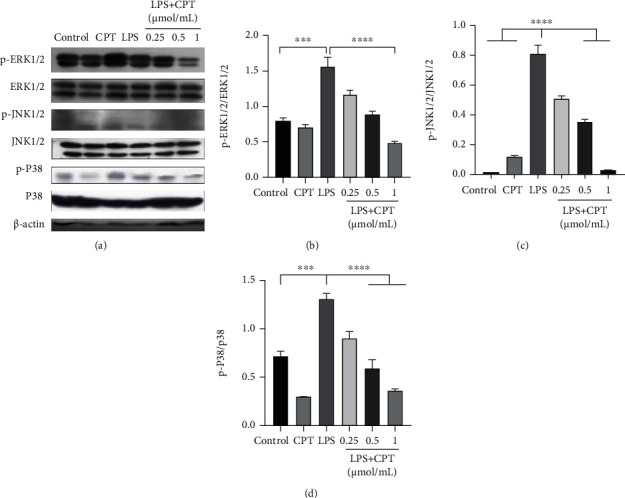
Effects of camptothecin (CPT) on LPS-induced activation of the MAPK signaling pathway in RAW264.7 cells. Total protein in RAW264.7 cells was collected after 4 h of LPS stimulation. CPT was added 1 h before LPS stimulation. Protein levels of (a, b) p-ERK1/2, (a, c) p-JNK1/2, and (a, d) p-P38 were detected by western blot and quantitatively assessed via densitometry using ERK1/2, JNK1/2, and P38 as internal controls. Protein levels were measured using ImageJ (http://imagej.nih.gov/ij/) and normalized to the protein levels of ERK1/2, JNK1/2, and P38. Values are presented as mean ± SD (^∗∗^*P* < 0.01 vs. LPS, ^∗∗∗∗^*P* < 0.0001 vs. LPS).

We investigated the signaling pathways activated by CPT for the purpose of further elucidating CPT's anti-inflammatory mechanism. First, we conducted pretreatment on these cells with CPT (0.25, 0.5, and 1.0 *μ*mol/mL). Then, we excited the cells with LPS (1 *μ*g/mL). After that, we detected the phosphorylation proteins. And inhibition effect was showed to be dose-dependent in CPT-inhibited LPS-induced phosphorylation proteins (Figures 7(a), 7(b), and Figures 8(a)–8(d)).

### 3.7. CPT Inhibits the Expression of the Transcription Factor p65 (or NF-*κ*B p65 Subunit)

**Figure 9:**
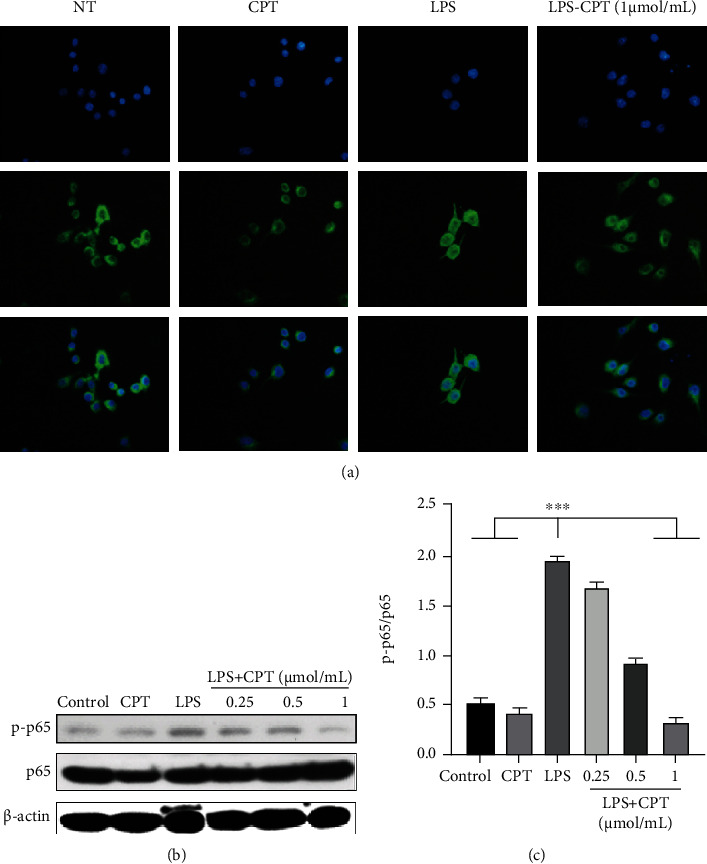
Effects of camptothecin (CPT) on the transcription factor p65 in the NF-*κ*B signaling pathway. RAW264.7 cells were induced by LPS, and the phosphorylation of p65 was assayed using immunofluorescence (a). Protein levels of p-p65 were detected by western blot and quantitatively assessed via densitometry using p65 as an internal control (b, c). The values presented are mean ± SD (^∗^*P* < 0.05, ^∗∗^*P* < 0.01, and ^∗∗∗∗^*P* < 0.0001 vs. LPS).

Immunofluorescence analysis was applied to show p65 expression in the NF-*κ*B signaling pathway under CPT effect. In resting cells, p65 binds to its inhibitory protein. Following LPS stimulation, p65 is released into the nucleus, resulting in a biological effect. The expression levels of p65 were remarkably significant in the LPS group (Figure 9(a)), whereas the p65 expression was significantly inhibited in the CPT groups (Figure 9(a)). Western blot analysis of p65 phosphorylation showed the dose-dependent inhibition of CPT on p65 phosphorylation which was induced by LPS (Figures 9(b) and 9(c)).

## 4. Discussion

UC is a chronic inflammatory disease whose main effects are concentrated in the colon. The incidence of UC is increasing globally [16]. Modern medical treatment is usually combined with drugs such as salicylates, glucocorticoids, and immunosuppressants. The clinical efficacy of these drugs in UC is unstable, and their administration causes side effects, for example, hepatorenal toxicity, drug dependence, and recurrence after hormone withdrawal. In this experiment, in order to simulate human UC, we constructed DSS-induced mouse enteritis model. Previous studies have shown that DSS-induced enteritis model has many similarities with human UC and can be used as a model for screening anti-UC drugs [17].

Current studies have shown that Chinese medicine monomer can play an important role in the treatment of colitis in mice. For example, Su et al. found that total matrines can inhibit the expression of cytokines such as TNF-*α* and IL-1*β*, thus reducing the inflammatory response of UC in rats [18]. Huang et al. found that curcumin significantly improved the symptoms and pathological morphology of UC in mice and inhibited the expression of TNF-*α* and IL-8 [19]. These studies revealed the important therapeutic functions and mechanisms of different monomers of traditional Chinese medicine (TCM) in UC from different perspectives [19, 20]. At present, there are relatively few studies on TCM monomers in the treatment of human ulcerative colon, but there are some TCM monomers that can be directly used in clinic. Therefore, the combination of TCM monomer and traditional UC drugs can obtain better clinical efficacy. This measure can significantly reduce the dosage and time of anti-inflammatory hormone and reduce the side effects of patients.

CPT is a TCM monomer with anti-inflammatory effect. Our study showed that CPT can effectively alleviate DSS-induced colitis in mice, reduce proinflammatory mediators in mice, and alleviate colon injury [17]. In in vitro experiments, we also fully confirmed that CPT can reduce the expression of proinflammatory mediator-related genes and proinflammatory enzymes in RAW264.7 [21]. At the same time, we verified the toxicity of different doses of CPT on RAW264.7 through the CCK8 test and found that large doses of CPT did not lead to cytotoxicity. This suggested that CPT can be used as a relatively safe drug for the treatment of UC.

Although CPT plays an important role in alleviating UC in mice, its mechanism is still unclear [22]. Current studies have shown that UC can activate proinflammatory signaling pathways in the body, leading to the increased phosphorylation of MAPKs and NF-*κ*B signaling pathway-related proteins [23, 24]. And our research also confirmed this. We detected the phosphorylation of MAPKs and NF-*κ*B signaling pathway-related proteins in vivo and in vitro. The results showed that CPT could significantly inhibit the phosphorylation of P38, ERK1/2, JNK1/2, and p65 as well as the nuclear transfer of p65. This suggested that CPT can inhibit the activation of the proinflammatory signaling pathway at the molecular level, which has the significance of further research and development.

## 5. Conclusion

Our results suggest that CPT improves UC which is induced by DSS in terms of colon length and the disease activity index in rats. Additionally, CPT inhibits the expressions of proinflammatory cytokines, which may be responsible for the phosphorylation of vital proinflammatory proteins in the AKT, NF-*κ*B, and MAPK signaling pathways. Thus, CPT is a potential drug for treating UC.

## Figures and Tables

**Table 1:** Disease activity index.

Score	Weight loss (%)	Stool appearance	Fecal occult blood
0	0	Normal form	Negative
1	1–5		
2	5–10	Loose stool	Positive
3	10–20		
4	>20	Diarrhea	Gross bleeding

**Table 2:** Primer sequences used to target *TNF-α*, *IL-1β*, *IL-6*, and *β-actin*.

Item	Primer	Amplicon length (bp)
TNF-*α* (sense)	5′-ACGGCATGGATCTCAAAGAC-3′	116
TNF-*α* (antisense)	5′-GTGGGTGAGGAGCACGTAGT-3′	
IL-1*β* (sense)	5′-GCTGCTTCCAAACCTTTGAC-3′	121
IL-1*β* (antisense)	5′-AGCTTCTCCACAGCCACAAT-3′	
IL-6 (sense)	5′-CCGGAGAGGAGACTTCACAG-3′	134
IL-6 (antisense)	5′-CAGAATTGCCATTGCACAAC-3′	
iNOS (sense)	5′-GAACTGTAGCACAGCACAGGAAAT-3′	158
iNOS (antisense)	5′-CGTACCGGATGAGCTGTGAAT-3′	
COX-2 (sense)	5′-CAGTTTATGTTGTCTGTCCAGAGTTTC-3′	127
COX-2 (antisense)	5′-CCAGCACTTCACCCATCAGTT-3′	
*β*-Actin (sense)	5′-GTCAGGTCATCACTATCGGCAAT-3′	147
*β*-Actin (antisense)	5′-AGAGGTCTTTACGGATGTCAACGT-3′	

## Data Availability

All data generated or analyzed during this study are included in this published article.

## Abbreviations

CPT: Camptothecin
TCM: Traditional Chinese medicine
UC: Ulcerative colitis
DSS: Dextran sodium sulfate
IL: Interleukin
AKT: Protein kinase B
NF-*κ*B: Nuclear factor-*κ*B
MAPK: Mitogen-activated protein kinase
LPS: Lipopolysaccharide
H&E: Hematoxylin and eosin
MPO: Myeloperoxidase
TNF: Tumor necrosis factor
ELISA: Enzyme-linked immunosorbent assay
iNOS: Inducible nitric oxide synthase
COX-2: Cyclooxygenase-2
ERK: Extracellular signal-regulated protein kinase
JNK: c-Jun N-terminal kinase
I*κ*B*α*: Inhibitor of nuclear factor-*κ*B
CCK8: Cell Counting Kit-8
qRT-PCR: Quantitative reverse transcription-polymerase chain reaction
IBD: Inflammatory bowel disease
FBS: Fetal bovine serum
DMEM: Dulbecco's modified Eagle's medium
HRP: Horseradish peroxidase
OD: Optical density
PBS: Phosphate-buffered saline
SDS: Sodium dodecyl sulfate.
